# Factors associated with health insurance ownership among women of reproductive age: A multicountry study in sub-Saharan Africa

**DOI:** 10.1371/journal.pone.0264377

**Published:** 2022-04-12

**Authors:** Liming Shao, Yiting Wang, Xuhui Wang, Lu Ji, Rui Huang

**Affiliations:** 1 Planning and Development Department, Hangzhou Medical College, Hangzhou, Zhejiang, China; 2 School of Business, Central South University, Changsha, China; 3 Hunan University of Finance and Economics, Changsha, China; 4 Guangdong Women and Children Hospital, Guangzhou, China; 5 School of medicine and health management, Tongji medical college, Huazhong University of Science and Technology, Wuhan, China; 6 School of Pharmacy, Tongji Medical College, Huazhong University of Science and Technology, Wuhan, China; Illawarra Shoalhaven Local Health District, AUSTRALIA

## Abstract

**Background:**

Promoting the coverage and ownership of health insurance constitutes a key strategy to achieving universal healthcare, thereby meeting the Sustainable Development Goal (SDG 3.8) of safeguarding the vulnerable population from financial risk resulting from catastrophic health expenditures. In sub-Saharan Africa, accessing medical services is particularly challenging among women due to inadequate opportunities for socio-economic empowerment and meeting their unique healthcare needs. The present study aimed to explore the sociodemographic factors associated with health insurance ownership among women in selected countries in sub-Saharan Africa.

**Methods:**

We extracted cross-sectional data on health insurance ownership and conceptually relevant sociodemographic variables on women aged 15–49 years from Demographic and Health Surveys on five selected countries in sub-Saharan Africa (n = 55,438), including Burkina Faso, DR Congo, Cameroon, Gabon and Kenya. Data were analyzed using descriptive and multivariate regression analysis, and the associations were reported in terms of average marginal effects.

**Results:**

Data revealed considerable cross-country variation in health insurance ownership: Gabon (57.9%), Kenya (6.1%), DR Congo (2.8%), Cameroon (1.1%), Burkina Faso (0.4%). In the multivariate regression analysis, women’s age, marital status, place of residency, educational level, household wealth status, employment, and media access were significant predictors of insurance ownership. The associations were generally similar across the five countries, with higher age, better educational level and wealth status showing a consistently positive relationship with insurance ownership.

**Conclusion:**

Our findings revealed striking inequality in health insurance ownership among the studied countries, which should be given due attention to achieving universal healthcare-related goals. There also exist considerable sociodemographic disparities in health insurance ownership among women that should be addressed using context-specific programmatic interventions. Our findings revealed striking inequality in health insurance ownership among the studied countries, which should be given due attention to achieving universal healthcare-related goals. There also exist considerable sociodemographic disparities in health insurance ownership among women that should be addressed using context-specific programmatic interventions.

## Introduction

During the last two decades, countries in sub-Saharan Africa have made substantial progress in critical public health indicators including reduction of maternal and child mortality [1–3]. Advancement in medical technologies, better health financing and global partnerships (such as MDGs and SDGs) have contributed to more efficient public health interventions and increased life expectancy [4–7]. Despite these achievements, the healthcare sector continues to face significant challenges in its efforts to ensure quality care for all citizens irrespective of their ability to pay for the services. This is particularly the case for women who experience more significant barriers to accessing essential healthcare services such as routine antenatal visits and childbirth services [8–13]. Arguably, innovation in health policy and the decision-making process in developing countries has been far outpaced by our growing understanding of the broader determinants of health and the development of life-saving technologies.

Due to the lack of appropriate institutional infrastructure to provide equitable and person-centred care, a significant proportion of the population continues to experience unmet needs for healthcare, with women and marginalized communities being disproportionately affected by this situation [14–16]. Secondly, although many countries have shown promising outcomes in improving several health-related indicators, they have lagged in others that function as distant but crucial determinants of women’s health and wellbeing [16]. As suggested by healthcare and social researchers, lack of empowerment opportunities such as access to quality education and social, political and labour market participation are preventing women from being able to make informed decisions about their health and other aspects of life that predict long-term health and outcomes (e.g. fertility, nutrition) [17–20]. Women are also more likely to experience catastrophic health expenditures, further worsening their empowerment capabilities and deepening impoverishment. Various measures have been proposed and taken to safeguard women from facing financial adversities and health risks, such as improved and free-of-charge access to maternity care under attaining universal healthcare (UHC), a vital component of the health-related Sustainable Development Goals (SDG) [21–25].

Since the adoption of the Sustainable Development Goals (SDG), there has been a renewed interest in the developing countries in reinforcing efforts to achieve universal health coverage to ensure equitable access to quality care among women. Specifically, target 3.8 of SDG is dedicated to safeguarding people from financial risk due to catastrophic health expenditures by minimizing out-of-pocket costs with the broader goal of improving population health and promoting socioeconomic well-being and national development at large [26–28]. Many countries have introduced national health insurance schemes to promote UHC. However, progress remains sub-optimal due to the lower adoption of insurance schemes in the population. Several studies have attempted to explore the factors associated with health insurance ownership, [29–32]. Still, they are primarily based on small-scale samples, and findings are not comparable across the studies due to methodological and measurement heterogeneities. In the present study, we aimed to address this gap by analyzing data from Demographic and Health Surveys that use uniform instruments for data collection. Demographic and Health Surveys are also nationally representative, which means that the generated estimations will be more reliable, generalizable for the entire population, and comparable across the analysis settings. Apart from the advantages of greater precision and power of the results, multicountry findings also allow cross-cultural comparison of the relationships between variables of interest. Insights developed from the present study are expected to inform population intervention programs that promote health insurance coverage by addressing the sociodemographic gaps.

## Methods

The present study was based on open-access data collected from Demographic and Health Surveys (DHS) in the following countries: Burkina-Faso (Survey year = 2010, n = 15,044, response rate = 98%), DR Congo (Survey year = 2013–14, n = 18,716, response rate = 99%), Cameroon (Survey year = 2011, n = 11,732, response rate = 97%), Gabon (Survey year = 2012, n = 6,067, response rate = 98.2%) and Kenya (Survey year = 2014, n = 20,964, response rate = 96%). These surveys are nationally representative and cover the community sample population, e.g. residing in households. We obtained data on adult women aged 15–49 years for this study. DHS surveys are conducted by collaboration by U.S. Agency for International Development (USAID), the United Nations Children’s Fund (UNICEF), and the United Nations Population Fund (UNFPA) with technical assistance for the survey was provided by ICF international. The main objectives of the survey were to collect data on key demographic indicators such as fertility, childhood mortality, maternal and child health status. Data serve the purpose of measuring the progress towards national and international development goals (such as Sustainable Development Goals) and facilitating evidence-based policies.

### Variables

The outcome variable was insurance ownership which was measured by asking the respondent about insurance ownership. Answer to this question was categorised as “Covered by health insurance” and “Not covered”. Several enabling and predisposing factors were chosen as the predictor variables based on their theoretical association with insurance ownership that was described in Table 1.

**Table 1:** Description of the variables included in the analysis.

Variables	Codebook	Description
*Outcome variable*		
Covered by health insurance	No (0), Yes (1)	Whether or not a respondent is currently insured
*Explanatory variables*		
Age	15–19, 20–24, 25–29, 30–34, 35–39, 40–44, and 45–49 years	Age of respondent at the time of survey
Marital status	Not married (1), Married (2)	Current marital status
Residency	Urban (1), Rural (2)	Type of place residency
Education	None/less than primary (0), Primary (1), Secondary (2), Higher (3)	Educational level based on total number of years of schooling
Wealth quintile	Poorest (1), Poorer (2), Middle (3), Richer (4), Richest (5)	Wealth index calculated based on passion of durable goods by a household
Employment	No (0), Yes (1)	Has outdoor employment
Media access	No (0), Yes (1)	Has access to TV and radio.

As explained in previous studies, wealth status is calculated by a score assigned to households based on the possession of durable goods (e.g., TV, radio, refrigerator, and construction material). Categorization was performed by dividing the household wealth scores into quintiles. The richest households were those in the highest quintile, and the poorest were in the lowest quintile [25].

### Data analysis

Data were analyzed with Stata version 16 (College Station, TX: Stata Corp LP). All analyses were adjusted for the cluster design by using the svy command. This command uses the information on sampling weight, strata, and primary sampling unit provided with the datasets. Sample characteristics were described as percentages with 95% confidence intervals. Percentages of respondents who reported having any health insurance were described separately for each country. Following that, binary logistic regression models were used to estimate the associations between health insurance ownership and the explanatory factors. Using the ‘margins’ functionality of Stata, these results were reported in terms of average marginal effects with 95% confidence intervals. At first, we ran a pooled model including all the countries, which was followed by a country-stratified analysis. Variance inflation factor (VIF) command was used to test for multi-collinearity. No multi-collinearity was detected as VIF values were below 10 for all the models. All tests were two-tailed and were considered significant at alpha value of 5%. Model Performance was assessed using the receiver operating characteristic curve (ROC curve). The final step of the analysis involved the calculation of percentage contribution of the variables to the total variance in the outcome factor for each of the five countries to highlight cross-country differences in the relative importance of the explanatory variables.

## Results

### Sample description

Sociodemographic characteristics of the sample participants were presented in Table 2. Overall, about in ten participants (9.9%, 95%CI=) reported having a health insurance. The relative percentage of having a health insurance was higher among those aged 40–44 years (12.2%, 95%CI = 11.1; 13.2), currently unmarried (20.3%, 95%CI=), urban resident (17.2%, 95%CI = 16.7; 17.8), had higher educational level (50%, 95%CI = 47.2; 52.9), from the richest wealth quintile households (16%, 95%CI = 15.1; 16.8), employed (15.1%, 95%CI = 14.5; 15.7), had access to electronic media (25.8%, 95%CI = 24.9; 26.7).

**Table 2:** Health insurance ownership by sociodemographic characteristics. (n = 55,438).

	N (%)	Covered by health insurance		
Variables		No	Yes	P-value
	55438 (100.0)	49940 (90.1%)	5498 (9.9%)	
**Age**				
15–19	6.2 (6.0; 6.4)	90.3 (89.3; 91.2)	9.7 (8.8; 10.7)	
20–24	23.4 (23.1; 23.8)	91.4 (91.0; 91.9)	8.6 (8.1; 9.0)	
25–29	27.7 (27.4; 28.1)	90.9 (90.5; 91.4)	9.1 (8.6; 9.5)	
30–34	20.2 (19.8; 20.5)	89.0 (88.5; 89.6)	11.0 (10.4; 11.5)	
35–39	13.9 (13.6; 14.2)	88.7 (88.0; 89.4)	11.3 (10.6; 12.0)	
40–44	6.8 (6.6; 7.0)	87.8 (86.8; 88.9)	12.2 (11.1; 13.2)	
45–49	1.8 (1.7; 1.9)	89.6 (87.7; 91.5)	10.4 (8.5; 12.3)	0.00
**Marital status**				
Not married	29.0 (28.6; 29.4)	79.7 (79.1; 80.4)	20.3 (19.6; 20.9)	
Married	71.0 (70.6; 71.4)	94.3 (94.1; 94.5)	5.7 (5.5; 5.9)	0.00
**Residency**				
Urban	32.3 (31.9; 32.7)	82.8 (82.2; 83.3)	17.2 (16.7; 17.8)	
Rural	67.7 (67.3; 68.1)	93.6 (93.3; 93.8)	6.4 (6.2; 6.7)	0.00
**Education**				
No education	36.9 (36.5; 37.3)	98.8 (98.7; 99.0)	1.2 (1.0; 1.3)	
Primary	36.3 (35.9; 36.7)	89.0 (88.6; 89.4)	11.0 (10.6; 11.4)	
Secondary	24.6 (24.2; 24.9)	82.1 (81.5; 82.7)	17.9 (17.3; 18.5)	
Higher	2.2 (2.1; 2.3)	50.0 (47.1; 52.8)	50.0 (47.2; 52.9)	0.00
**Wealth quintile**				
Poorest	27.7 (27.4; 28.1)	86.3 (85.8; 86.8)	13.7 (13.2; 14.2)	
Poorer	21.8 (21.5; 22.2)	92.8 (92.3; 93.2)	7.2 (6.8; 7.7)	
Middle	19.8 (19.4; 20.1)	93.9 (93.5; 94.3)	6.1 (5.7; 6.5)	
Richer	17.3 (17.0; 17.7)	93.0 (92.5; 93.5)	7.0 (6.5; 7.5)	
Richest	13.3 (13.0; 13.6)	84.0 (83.2; 84.9)	16.0 (15.1; 16.8)	0.00
**Employment**				
No	25.7 (25.3; 26.0)	91.9 (91.6; 92.1)	8.1 (7.9; 8.4)	
Yes	74.3 (74.0; 74.7)	84.9 (84.3; 85.5)	15.1 (14.5; 15.7)	0.00
**Media access**				
No	71.1 (70.6; 71.6)	95.8 (95.5; 96.0)	4.2 (4.0; 4.5)	
Yes	28.9 (28.4; 29.4)	74.2 (73.3; 75.1)	25.8 (24.9; 26.7)	0.00

N.B. For total sample column percentage was reported. For health insurance, row percentage was reported.

**Fig 1:**
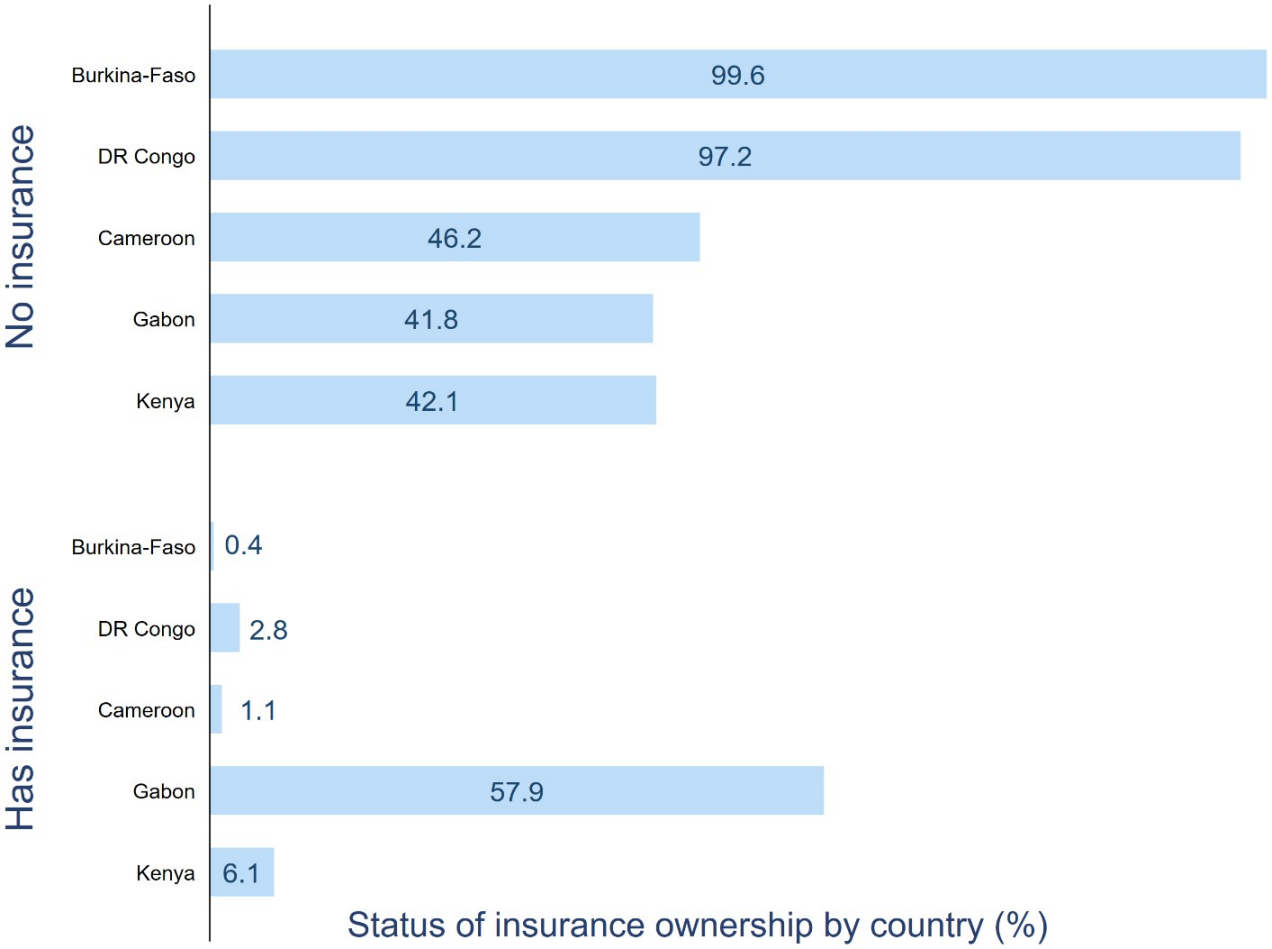
Percentage of participants with a health insurance by country.

From Fig 1, the highest percentage of insurance ownership was observed for Gabon with more than half of the participants reporting having a plan (57.9%), and lowest for Burkina Faso (0.4%). These cross-country differences in insurance ownership were (P<0.001) statistically significant.

**Fig 2:**
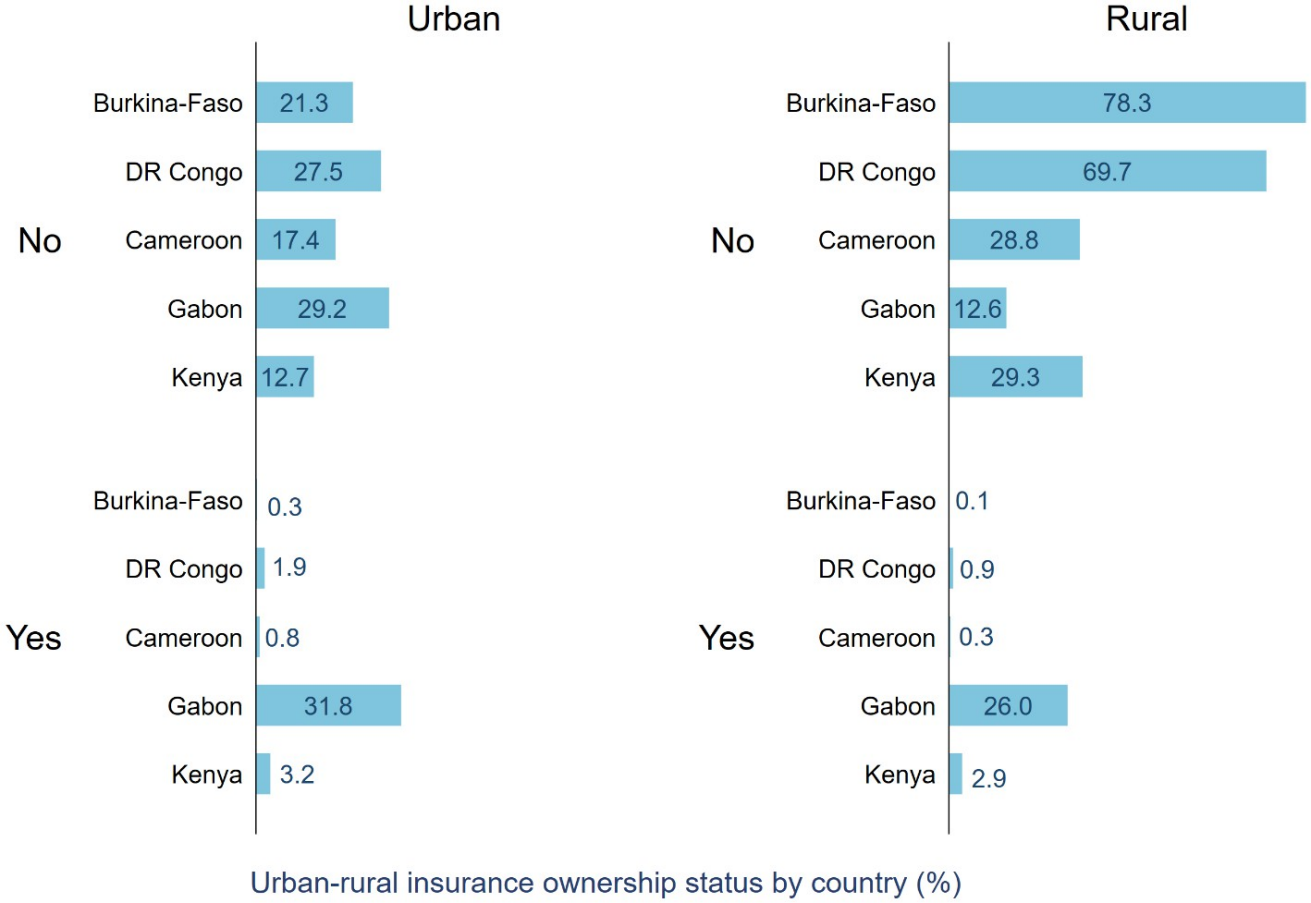
Percentage of participants with health insurance in urban and rural areas.

We further compared the differences in health insurance ownership between rural and urban residents. From Fig 2 it is evident that the percentage of insurance ownership was higher among urban women in all countries. These regional differences in insurance ownership were (P<0.001) statistically significant as well.

The results of multivariate logistic regression calculating the association between health insurance ownership with the sociodemographic factors were presented in Table 3. For the overall sample, we observed an increasingly higher likelihood of insurance ownership for higher age groups. For instance, participants in the age groups of 40–44 years and 45–49 years had 11% and 8% higher likelihood of having a plan respectively. After stratifying for countries, the age difference remained significant for Gabon and Kenya only where the likelihood was 37% and 13% higher respectively among those in the age group of 40–44 years. Marital status showed an inverse association with having plan for the overall sample (AME = 0.94, 95%CI = 0.94,0.95) and for Gabon (AME = 0.92, 95%CI = 0.88,0.97), while in Kenya it showed a positive association (AME = 1.05, 95%CI = 1.03, 1.07). Rural residency (AMR = 0.97, 95%CI = 0.96,0.98) also showed an inverse association in the overall sample, but the association didn’t hold for country-specific analysis. Participants in the higher educational categories were more likely to have a plan in the overall (AMR = 1.41, 95%CI = 1.37,1.45) and individual countries except for DR Congo. Positive effect on having a plan was observed for all higher quantiles of wealth as well. For instance, 65% (95%CI = 1.12,2.42) and 23% (95%CI = 1.18,1.28) higher likelihood was observed in Gabon and Kenya respectively. Having employment showed a lower likelihood of plan ownership in the overall sample but positive in other countries except for DR Congo. Finally, having access to electronic media showed strong positive association with plan ownership in the overall sample (AME = 1.20, 95%CI = 1.18,1.21), and in Burkina Faso (AME = 1.03, 95%CI = 1.01,1.04), DR Congo (AME = 1.03,1.01, 95%CI = 1.04) and Kenya (AME = 1.04, 95%CI = 1.01,1.07).

**Table 3:** Results of multivariate logistic regression estimations of the proportions of insurance ownership regressed on the sociodemographic factors in five SSA countries.

	Overall	Burkina-Faso	DR Congo	Cameroon	Gabon	Kenya
**Age (15–19)**						
20–24	1.01 (1.00,1.02)	0.98 (0.95,1.02)	0.99 (0.98,1.00)	0.98 (0.94,1.01)	1.08** (1.02,1.15)	1.05 (1.00,1.10)
25–29	1.03*** (1.02,1.04)	0.98 (0.94,1.02)	0.99 (0.98,1.01)	0.97 (0.94,1.01)	1.15*** (1.08,1.22)	1.09*** (1.05,1.15)
30–34	1.06*** (1.05,1.08)	0.98 (0.95,1.02)	1.00 (0.99,1.02)	0.99 (0.95,1.03)	1.24*** (1.16,1.32)	1.11*** (1.05,1.16)
35–39	1.09*** (1.07,1.10)	0.99 (0.95,1.03)	1.00 (0.99,1.02)	1.01 (0.97,1.06)	1.29*** (1.21,1.38)	1.11*** (1.06,1.17)
40–44	1.11*** (1.09,1.12)	0.98 (0.94,1.02)	1.01 (0.99,1.03)	0.99 (0.94,1.04)	1.37*** (1.27,1.47)	1.13*** (1.06,1.20)
45–49	1.08*** (1.05,1.11)	1.05 (0.94,1.17)	0.99 (0.97,1.02)	1.02* (1.00,1.04)	1.21** (1.07,1.37)	1.06 (0.97,1.16)
**Marital status** (Single)						
Married	0.94*** (0.94,0.95)	1.01 (1.00,1.02)	1.01*** (1.01,1.02)	1.01 (1.00,1.02)	0.92*** (0.88,0.97)	1.05*** (1.03,1.07)
**Residency (Urban)**						
Rural	0.97*** (0.96,0.98)	1.00 (0.99,1.01)	1.01 (1.00,1.02)	1.01 (0.99,1.03)	1.04 (1.00,1.08)	1.00 (0.98,1.02)
**Education (No education)**						
Primary	1.08*** (1.08,1.09)	1.01 (1.00,1.01)	0.98 (0.97,1.04)	1.03** (1.01,1.05)	1.28*** (1.18,1.37)	1.03 (1.00,1.06)
Secondary	1.12*** (1.11,1.13)	1.03*** (1.01,1.06)	0.99 (0.98,1.01)	1.01 (1.00,1.03)	1.22*** (1.14,1.32)	1.10*** (1.06,1.13)
Higher	1.41*** (1.37,1.45)	1.23*** (1.09,1.38)	1.02 (1.00,1.04)	1.09*** (1.05,1.14)	1.39*** (1.25,1.54)	1.30*** (1.24,1.37)
**Wealth quintile (Poorest)**						
Poorer	1.07*** (1.04,1.10)	1.02 (0.99,1.05)	0.99** (0.99,1.00)	1.16*** (1.11,1.20)	1.25*** (1.16,1.35)	1.06*** (1.03,1.09)
Middle	1.11*** (1.08,1.15)	1.06*** (1.03,1.09)	1.00 (0.99,1.00)	1.19*** (1.14,1.24)	1.32*** (1.18,1.48)	1.11*** (1.08,1.15)
Richer	1.12*** (1.08,1.16)	1.09*** (1.05,1.12)	1.01** (1.00,1.02)	1.17*** (1.12,1.23)	1.46*** (1.23,1.75)	1.16*** (1.13,1.20)
Richest	1.17*** (1.11,1.22)	1.09*** (1.05,1.13)	1.10*** (1.06,1.14)	1.19*** (1.12,1.27)	1.64* (1.12,2.42)	1.23*** (1.18,1.28)
**Has employment (No)**						
Yes	0.97*** (0.96,0.98)	1.01* (1.00,1.02)	1.00 (1.00,1.01)	1.03*** (1.01,1.04)	1.04* (1.01,1.08)	1.05*** (1.03,1.07)
**Access to media (No)**						
Yes	1.20*** (1.18,1.21)	1.03*** (1.01,1.04)	1.03*** (1.01,1.04)	1.02 (1.00,1.04)	0.97 (0.93,1.02)	1.04* (1.01,1.07)
***Pseudo-r2***	***0*.*312***	***0*.*210***	***0*.*227***	***0*.*249***	***0*.*205***	***0*.*330***

N.B. Numbers represent average marginal effects with 95% confidence intervals in parenthesis. Level of significance:

* *p* < 0.05,

** *p* < 0.01,

*** *p* < 0.001.

**Fig 3:**
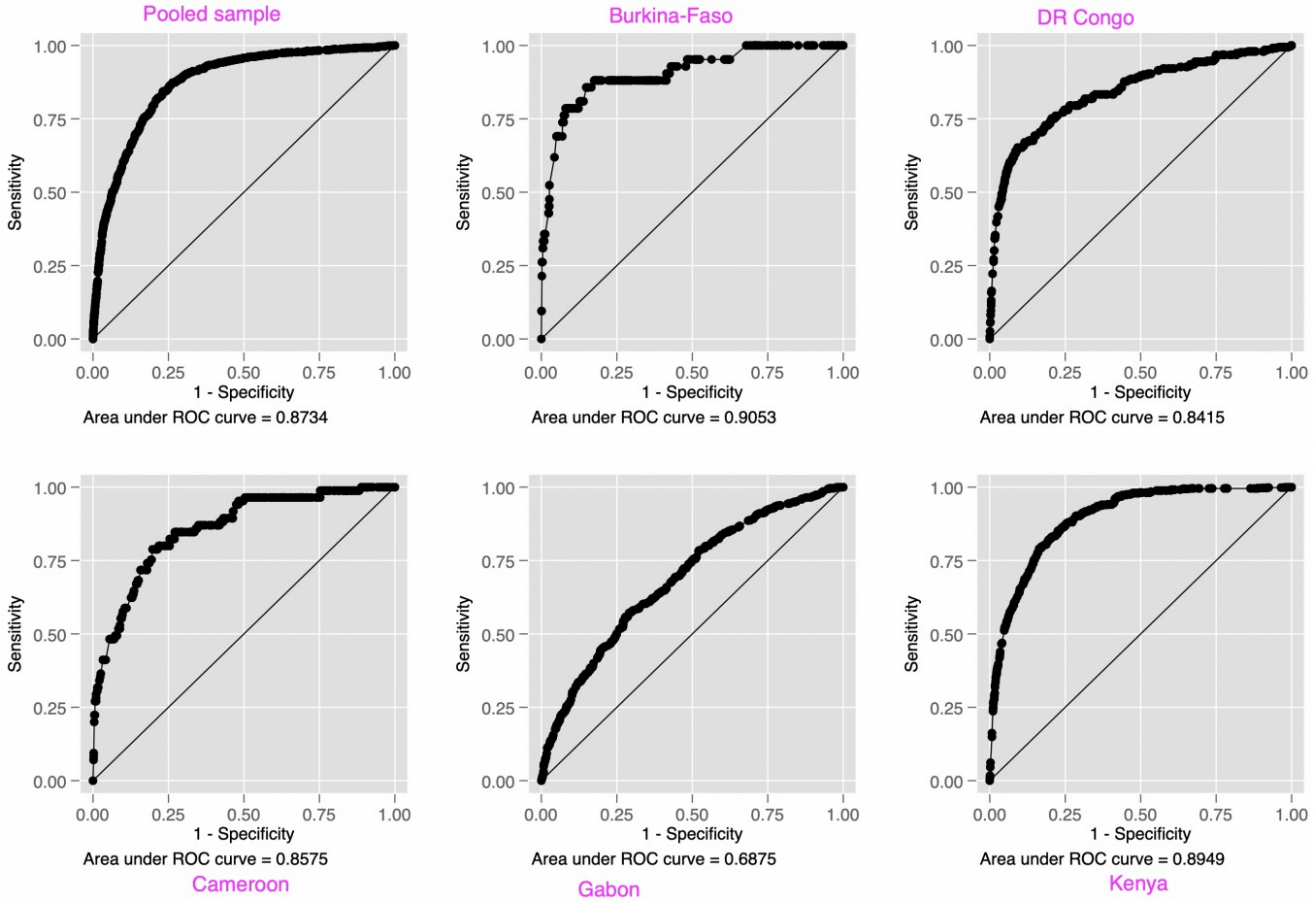
Receiver operating curves.

Following the regression analyses, we constructed receiver operating characteristic (ROC) curve to assess the predicting power of the models for the pooled sample and the individual countries as well. As shown in the Fig 3, the ROC curve value was ranged from 0.69 to 0.90 which is considered moderate to good [33].

**Fig 4:**
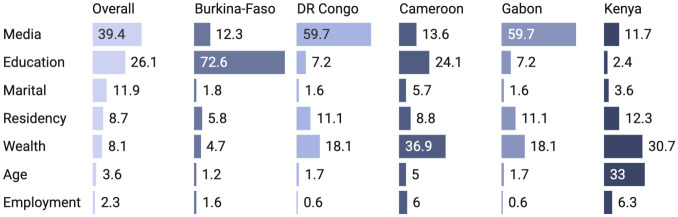
Relative importance of the variables in the equations.

Fig 4 shows the relative importance of the variable in explaining the total variance in insurance ownership in terms of percentage contributions. As indicated by the chart, access to media was the largest contributor to insurance ownership in the overall sample, as well as in DR Congo and Gabon. For Burkina Faso, education was the most important predictor (72.6% variance) compared with wealth status (36.9%) in Cameroon and age (33%) in Kenya.

## Discussion

Using cross-sectional data from Demographic and Health Surveys, the present study reports the percentage of health insurance ownership and its sociodemographic correlates among adult women aged between 15–49 years in Burkina-Faso, DR Congo, Cameroon, Gabon, and Kenya [34]. Healthcare delivery system in all five countries is characterized by similar challenges including lack of skilled health professionals, underdeveloped infrastructure and inequality in access to care. Our findings indicated that there exist great disparities in the percentage of insurance ownership among women in these five countries ranging from as high as 57.9% in Gabon to as low as 0.4% in Burkina Faso.

The noticeably higher percentage of insurance ownership in Gabon is understandable given the efforts made by the country to bring every citizen under one social insurance scheme called ‘Caisse Nationale d’Assurance Maladie et de Garantie Sociale (CNAMGS) [35]. Gabon also has the smallest population among the five countries and the highest per capita GDP which might potentially explain the noticeably higher percentage of insurance ownership among the five countries. Funding social insurance programs are challenging for low-income countries which must be dealt with innovative heath financing strategies which can pay off in the long term through lowering healthcare costs.

Further descriptive analysis revealed a significant urban-rural disparity in insurance ownership. In all five countries, participants in the rural areas had a lower percentage of having insurance, which is in line with the existing evidence regarding the greater disadvantages rural populations face in accessing healthcare. Our findings indicate significant between- and within-country disparities in health insurance ownership among women and call for intervention programs to address them. While interpreting these statistics, it is important to bear in mind that our sample population included only women and the age group of 15–49 years, and therefore the percentage is not representative of the overall population. The relatively younger age range could also explain in part the lower percentage of insurance ownership and its associated factors such as access to resources to enter the labour market.

Results of the multivariate regression analysis provided a more detailed scenario of the sociodemographic differences in insurance ownership. In short, women’s age, marital status, place of residency, educational level, household wealth status, employment, and media access were found to be significantly associated with insurance ownership. In general, higher age, better educational level and wealth status showed a consistently positive relationship with insurance ownership. These findings reflect the evidence from previous studies that showed a positive association between socioeconomic status and insurance ownership [26, 30, 31, 36]. Individuals with higher educational status and financial standing are generally more likely to be aware of potential health-related expenditure and capable of affording insurance plans. It is therefore of utmost importance that socioeconomic disparities in insurance ownership be reduced among women to ensure equitable access to essential healthcare services, e.g. sexual and reproductive healthcare. Finally, we calculated the relative importance of the predictor variables in the equation which captured the variation in the significance of the individual factors in insurance ownership in each country. Varying degrees (in terms of percentage contribution to the outcome factor) of the contribution of the individual factors may imply that the countries are different in terms of the contextual factor that determines insurance ownership, and that uniform policies or programs targeting those factors may not produce desired outcomes. In other words, context-oriented and locally-tailored interventions are necessary to improve insurance ownership.

In sub-Saharan Africa, the barriers to accessing medical services are particularly challenging among women due to their inadequate socioeconomic empowerment opportunities and meeting their special healthcare needs. Promoting labour market participation among women and ensuring employment insurance benefits can prove beneficial in this regard. Another important finding was the positive association between having access to electronic media and plan ownership. It is worthy of mentioning that this association was statistically significant for the overall sample and in Burkina Faso DR Congo and Kenya only. These cross-country comparisons of the predictors of insurance ownership highlight the importance of taking local contexts into account when designing health programs.

This study contributes to the literature on health insurance ownership among women in selected countries in sub-Saharan Africa. Data on demographics and other critical health indicators are extremely scarce for most countries in the African continent. From this perspective Demographic and Health Surveys provide a crucial source to fill this data gap and facilitate evidence-based population health planning and intervention in the beneficiary countries. This has several strengths that are worthy of mentioning. The datasets contained large sample size and are nationally-representative. Therefore, the findings are generalisable for the entire women population. In low-income settings, gathering data on large sample is constrained by budgetary limitations and lack of adequate research infrastructure. Furthermore, small-scale studies are generally conducted in line with the local circumstances and are usually not comparable to those from other settings due to variations in methodological approach and measurements of variables. From this viewpoint, our study has an additional advantage since measurements are homogenous in structure and measurements are also standardised to allow cross-cultural comparison.

Despite these advantages, there are some important limitations that are associated with the data. First, these are cross-sectional surveys, and therefore our analyses cannot guarantee any causal relationship between the outcome and explanatory factors. Since the surveys are secondary, the choice of the variables was contingent on their availability in the datasets. There were also no details regarding the types of insurance schemes owned by the participants such as whether or not they are public or private, source of funding, and the types of services they covered. Further studies need to conducted to address these limitations.

## Conclusion

This was a cross-sectional study of Demographic and Health Surveys, and the aim was to report the percentage of health insurance ownership and its sociodemographic correlates among adult women aged between 15–49 years in Burkina-Faso, DR Congo, Cameroon, Gabon, and Kenya. Women’s health is an underappreciated area of research in sub-Saharan Africa, and therefore studies of this kind can make important contributions to the advancement of health programs including addressing the disparities in the coverage of health insurance ownership. Our findings showed striking inequality in health insurance ownership among these five countries which should be given due attention for promoting universal healthcare-related goals. Based on these findings, it is suggestible that health policymakers take concrete steps to minimize the sociodemographic inequalities in health insurance ownership among women by taking into account the country-specific contextual factors.
